# Personality dimensions emerging during adolescence and young adulthood are underpinned by a single latent trait indexing impairment in social functioning

**DOI:** 10.1186/s12888-018-1595-0

**Published:** 2018-01-26

**Authors:** Ela Polek, Peter B. Jones, Pasco Fearon, Jeannette Brodbeck, Michael Moutoussis, NSPN Consortium, Ray Dolan, Peter Fonagy, Edward T. Bullmore, Ian M. Goodyer

**Affiliations:** 1 Department of Psychiatry, University of Cambridge, Herchel Smith Building, Forvie Site, Hills Road, Cambridge, CB2 0SZ UK; 2 School of Psychology, University College, Dublin, Ireland; 3 NIHR Collaboration for Leadership in Applied Health Research & Care East of England and NIHR Cambridge Biomedical Research Centre, Cambridge, CB2 8AH UK; 4 Division of Psychology and Language Sciences, University College London, 19 Torrington Place, London, WC1E 7HB UK; 5 Department of Clinical Psychology and Psychotherapy, University of Berne, 8 Fabrikstrasse, 3012 Bern, Switzerland; 6 Wellcome Trust Centre for Neuroimaging, University College London, 12 Queen Square, London, WC1N 3BG UK; 7 Wellcome Trust Centre for Neuroimaging, University College London, London, UK; 8 Division of Psychology and Language Sciences, University College London, Gower Street, London, WC1E 6BT UK; 9 Department of Psychiatry, University of Cambridge, Douglas House, 18b Trumpington Road, Cambridge, CB2 8AH UK

**Keywords:** Personality, Adolescence, Young adulthood, Schizotypal, Narcissistic, Callous, Unemotional, Antisocial, Negative emotionality, Impulsivity

## Abstract

**Background:**

Personality with stable behavioural traits emerges in the adolescent and young adult years. Models of putatively distinct, but correlated, personality traits have been developed to describe behavioural styles including schizotypal, narcissistic, callous-unemotional, negative emotionality, antisocial and impulsivity traits. These traits have influenced the classification of their related personality disorders. We tested if a bifactor model fits the data better than correlated-factor and orthogonal-factor models and subsequently validated the obtained factors with mental health measures and treatment history.

**Method:**

A set of self-report questionnaires measuring the above traits together with measures of mental health and service use were collected from a volunteer community sample of adolescents and young adults aged 14 to 25 years (*N* = 2443). *Results:* The bifactor model with one general and four specific factors emerged in exploratory analysis, which fit data better than models with correlated or orthogonal factors. The general factor showed high reliability and validity.

**Conclusions:**

The findings suggest that a selected range of putatively distinct personality traits is underpinned by a general latent personality trait that may be interpreted as a severity factor, with higher scores indexing more impairment in social functioning. The results are in line with ICD-11, which suggest an explicit link between personality disorders and compromised interpersonal or social function. The obtained general factor was akin to the overarching *dimension of personality functioning* (describing one’s relation to the self and others) proposed by DSM-5 Section III.

**Electronic supplementary material:**

The online version of this article (10.1186/s12888-018-1595-0) contains supplementary material, which is available to authorized users.

## Background

Adolescence and young adulthood is a critical period of maturation when stable behavioural styles emerge that pave the way for personality traits and related psychopathologies in adulthood. In particular, developmental changes occurring during this maturation process are important for the emergence of personality difficulties that involve antisocial behaviours and interpersonal relating. In this study we focused on negative emotionality (a long-term propensity to experience negative emotions), schizotypal trait (a pervasive pattern of interpersonal deficits marked by social anxiety and reduced capacity for close relationships as well as by cognitive or perceptual distortions and eccentricities of behaviour), narcissistic trait (a long-term pattern of exaggerated feelings of self-importance, an excessive need for admiration, and a lack of understanding of others’ feelings), callous-unemotional trait (a persistent pattern of behaviour that reflects a disregard for others, a lack of empathy and generally deficient affect), antisocial trait (a long-term pattern of manipulating, disregarding and exploiting others) and impulsivity trait (a tendency to display little or no forethought, or consideration of the consequences of one’s behaviour).

Although all the above traits have common defining features related to deficiencies in social and emotional functioning (e.g., difficulties in confiding and forming close and stable relations with others, suspiciousness, low trust, negative and unstable affect) [1–4], they were rarely studied together, thus empirical evidence on how they correlate is limited. However, the existing research suggests that these traits may share a considerable amount of variance. For example, in the study on two samples of 247 college students and 225 community residents, in which oversampling of individuals with schizotypal trait was used, schizotypal trait was associated with higher negative affect and lower clarity of emotions [5]. In a study of 50 student volunteers schizotypal trait was associated with impaired facial affect recognition [6]. Similar results regarding impaired emotional functioning were obtained for antisocial personality, callous-unemotional and impulsivity trait in a sample of 55 males [7, 8]. Volatile and negative affect have been associated with impulsivity in a sample of 481 college students [9]. Altered – higher or lower – levels of anxiety were related to antisocial traits in a community-based sample of 391 children [10] and higher anxiety was found to be related to schizotypy in the sample of 3807 university students [11]. Moreover, callous-unemotional, antisocial, narcissistic and impulsivity traits were found correlated in a sample of 720 adolescents (69% males) [12]. Impulsivity was also associated with schizotypal traits in 101 community adolescents [13].

There is evidence that both genetic [14] and neural features [15, 16] may be shared by putatively distinct personality traits. In line with this evidence, psychometric studies show that questionnaire dimensions of externalising and internalising difficulties are underlined by a common latent factor [17], and that various personality disorders and symptoms of mental health disorders may be underlined by one latent dimension [18, 19]. Therefore, we reasoned that, contrary to the generally accepted models of personality comprised of correlated dimensions, bifactor models of personality may better fit empirical data. There is still no published research investigating bifactor models using questionnaire assessment of personality traits in a general population and in particular adolescents and young adults, except for studies conducted on clinical adult populations [18]. Yet, the dimensional approach to personality disorders proposed in DSM-5 Section III implies continuity of personality traits between clinical and general populations, thus demanding evidence on validity of bifactor models in healthy populations.

### Aim and hypotheses

In this study, we aimed to reveal if a bifactor model would provide a better fit to the data compared to models comprised of orthogonal factors or correlated factors (hypothesis 1). Moreover, we hypothesised (hypothesis 2) that general and specific factors within a bifactor model would capture meaningful psychological constructs (as revealed in validity analyses). In particular, we expected a putative general factor to relate to the overarching construct describing *the level of personality functioning* proposed by DSM-5 Section III [20]. In addition, we aimed to test age and gender effects on levels of general and specific personality factors in emerging adults which, to our knowledge, have not been studied before. These effects (reported in the Additional file 1) may reveal developmentally sensitive period for the occurrence of prodromal signs of personality and mental health disorders in boys and girls.

## Method

### Participants

We used the data from the first wave of the Neuroscience in Psychiatry Network (NSPN) 2400 Cohort; a volunteer, community-based longitudinal sample of young people living in the area of Cambridgeshire and Greater London, UK [21]. Postal invitations to participants were sent through general practitioners and schools. Leaflets about the study were handed out in secondary schools, colleges and health centres. Invitation to participate was also placed on the NSPN website. Participants returned the expression of interest by post or by email to the study team. Written informed consent was obtained for all participants over the age of 16 and written consent from a parent/legal guardian was obtained for younger participants together with their assent. Using purposive sampling to obtain at least 200 males and females in 5 age groups (14–15, 16–17, 18–19, 20–21, 22–24), the questionnaire pack was sent to 3726 participants (and to parents of those under 18 years), who expressed initial interest. It was returned by 65% of them (*N* = 2403). In order to increase the power of validation analysis, 40 additional participants (aged 14–17) were recruited at the community treatment centres. Hence, the effective sample in this study was 2443 (54% female; 41% living in the Greater London Area; 59% living in Cambridgeshire). The socioeconomic profile of the NSPN 2400 Cohort approximated that of the population in England and Wales [21].

The study was carried out in accordance with the Declaration of Helsinki and Good Clinical Practice guidelines. Ethical approval was granted by the National Health Service Research Ethics Committee (project ID 97546).

### Measures

The questionnaire used in the study comprised a demographic section asking about date of birth (used for coding age), gender (here coded as: 1-male, 0-female). There were 122 items from five questionnaires, briefly described below (all items used in the bifactor model are listed in the Additional file 1). Six items were excluded as unsuitable for participants living with their parents and attending school or college (e.g., “I change jobs” or “I change residences”). The 24-item Inventory of Callous and Unemotional Traits (ICU [22]) had a response scale from not at all (0) to definitely true (3) and 3 subscales measuring callous-unemotional and antisocial traits: Unemotional (5 items), Callousness (11 items), Uncaring (8 items). The 17-item Antisocial Process Screening Device (APSD [23]) had a response scale from not at all true (0) to definitely true (2) and 3 subscales measuring narcissistic, callous-unemotional traits and impulsivity: Narcissism (7 items), Impulsivity (5 items), Callous-Unemotional (5 items). The 27-item Child and Adolescent Dispositions Scale (CADS [24]) had a response scale from not at all (1) to very much (4) and 3 subscales measuring negative emotionality, risk taking and antisocial traits: Negative Emotionality (9 items), Daring (5 items), Prosociality (13 items). The 25- item Barratt Impulsiveness Scale (BIS [25]) measuring various aspects of impulsivity trait had a response scale from rarely (1) to always (4) and 3 subscales: Nonplanning (11 items), Motor Impulsiveness (8 items), Attentional Impulsiveness (7 items). The 22-item Schizotypal Personality Questionnaire (SPQ [26]) had binary response options: yes (1) and no (0), and 3 subscales measuring aspects of schizotypal trait related to cognitive and perceptual idiosyncrasies, social avoidance and social anxiety, and disorganised behaviour: Cognitive-Perceptual (8 items), Interpersonal (8 items), Disorganised (6 items).

The following measures were used as external validation criteria: the Cambridge Friendships Questionnaire (CFQ [27]); the Moods and Feelings Questionnaire (MFQ [28]); the Revised Children’s Manifest Anxiety Scale (RCMAS [29]); the Revised Leyton Obsessional Inventory (R-LOI [30]); the Antisocial Behaviour Questionnaire (ABQ [31]); the Rosenberg Self-Esteem Scale (RSE [32]); the Warwick-Edinburgh Mental Well-being Scale (WEMWBS [33]), and also a retrospective measure of childhood experiences of parenting practices – the subscale of Positive Parenting from the Alabama Parenting Questionnaire (APQ [34])*.*

Two questions “*Are you currently being treated for any emotional, behavioural or mental health problems? Have you had any similar problems in the past?”* with answering options *yes* (1) and *no* (0) were used to identify individuals who were currently and/or in the past treated for mental health problems (coded as a binary variable: (1) past and/or present treatments, (0) no treatments). A question about the frequency of non-suicidal self-harm was asked “*In the last year, have you tried to hurt yourself on purpose without trying to kill yourself?”.* Answers ranging from *never* to *every day, or nearly every day* were used to derive a binary indicator coded as (0) no self-harm, (1) self-harm.

### Analytical strategy

Correlations between 116 items used in bifactor analysis were computed in *R qgraph* and visualised as a network plot (see the Additional file 1, Fig. 1). Confirmatory factor analyses with Mplus 7.4 of the original questionnaires showed moderate to low fit indices of the models; several items had very low (below .25) loadings on their original scales. However, we retained these items because they could still perform well in the bifactor model. Due to poor performance of original scales, an exploratory approach was taken and a method proposed by Preacher and colleagues [35] was used to determine the number of factors. They demonstrated that a cut-off of point of 0.05 for RMSEAs is indicative of the most replicable factorial solution in terms of a number of emerging factors. Thus, in the first step we computed in Mplus 7.4 a series of EFAs (Exploratory Factor Analysis) with WLSMV estimator and increasing number of factors to find a factorial solution meeting this criterion.

We further examined for evidence of a single general latent factor underlying all items by computing in Mplus 7.4 a unidimensional model (U), and if extraction of specific factors (in addition to a general factor) was necessary. The latter was determined by comparing fit of the unidimensional (U) and the bifactor model (C). Subsequently, we computed three EFA models with ML estimator (with number of factors indicated by the results of EFA with WLSMV estimator in the first step) and we used (A) orthogonal Geomin, (B) oblique Geomin, and (C) bi-Geomin orthogonal (bifactor) rotations, respectively. Based on the results of these three EFAs we computed three CFA (Confirmatory Factor Analysis) models where (A) factors were modelled as orthogonal and items were assigned to factors based on EFA with orthogonal Geomin rotation, (B) factors were modelled as correlated and items were assigned to factors based on EFA with oblique Geomin rotation, (C) factors were modelled as orthogonal and all items were assigned to load on one general factor, in addition to some items being assigned to load on specific factors based on EFA with bi-Geomin rotation. In all CFAs an item was assigned to a given factor, if the value (positive or negative) of their loading on this factor was .30 or above, in a respective EFA preceding each CFA. If an item had more than one loading with a value above .30, then the highest loading was used to determine item assignment to a factor. The three CFA models – A, B and C – were then compared using Akaike Information Criterion (AIC) [36], Bayesian Information Criterion (BIC) [37] and BIC adjusted for sample size (SABIC) [38]. These goodness-of-fit indices inform about the adequacy of a model in terms of fitting data and model parsimony, with lower values indicating the best balance between fit and parsimony.

When questionnaire data have a multidimensional structure (in particular with correlated factors), the standard practice is to report coefficient Alpha for a total scale and for subscales. However, in the case of a bifactor model omega coefficients are reported instead of Alpha: omega hierarchical (ω_H_), omega general (ω_G_) and omega specific (ω_S_) [39]. Formulae used to compute these coefficients and their definitions were reported in the Additional file 1.

To examine validity of obtained dimensions we computed in IBM SPSS 22 nonparametric correlations (Spearman rho) between factor scores (computed in Mplus 7.4 in bifactor CFA with one general and 4 specific factors) and external validation criteria: conduct problems, depression, anxiety, obsessionality, self-esteem and well-being as well as a retrospective measure of parenting experiences, a measure of quality of friendships, a measure of self-harm and current and/or past treatment for any emotional or mental health problems.

The Item Response Theory (IRT) framework (computed in bifactor CFA in Mplus 7.4) was utilised to examine conditional standard error of measurement for obtained factors. The data for conditional standard errors for each factor were exported from Mplus output and plotted in Excel for Windows. The increase in the curve depicting conditional standard error of measurement (see Fig. 2), which is usually occurring on both ends on the distribution, can be interpreted as higher measurement error for individuals with more extreme (higher and lower) scores on a latent dimension.

Univariate General Linear Model with Bonferroni post-hoc test and correction for multiple comparisons was computed in IBM SPSS 22 to estimate main and interaction effects of age and gender on levels of latent traits (indicated by factor scores). In the Additional file 1 we reported mean differences and effect sizes indicated by partial eta squared informing about the amount of variance in a dependent variable accounted for by a predictor when other effects are accounted for. To aid the interpretation of these effects we plotted age and gender effects on factor scores with 95% confidence intervals in Stata 14 using *twoway* command (Fig. 2 in the Additional file 1).

## Results

**Fig. 1:**
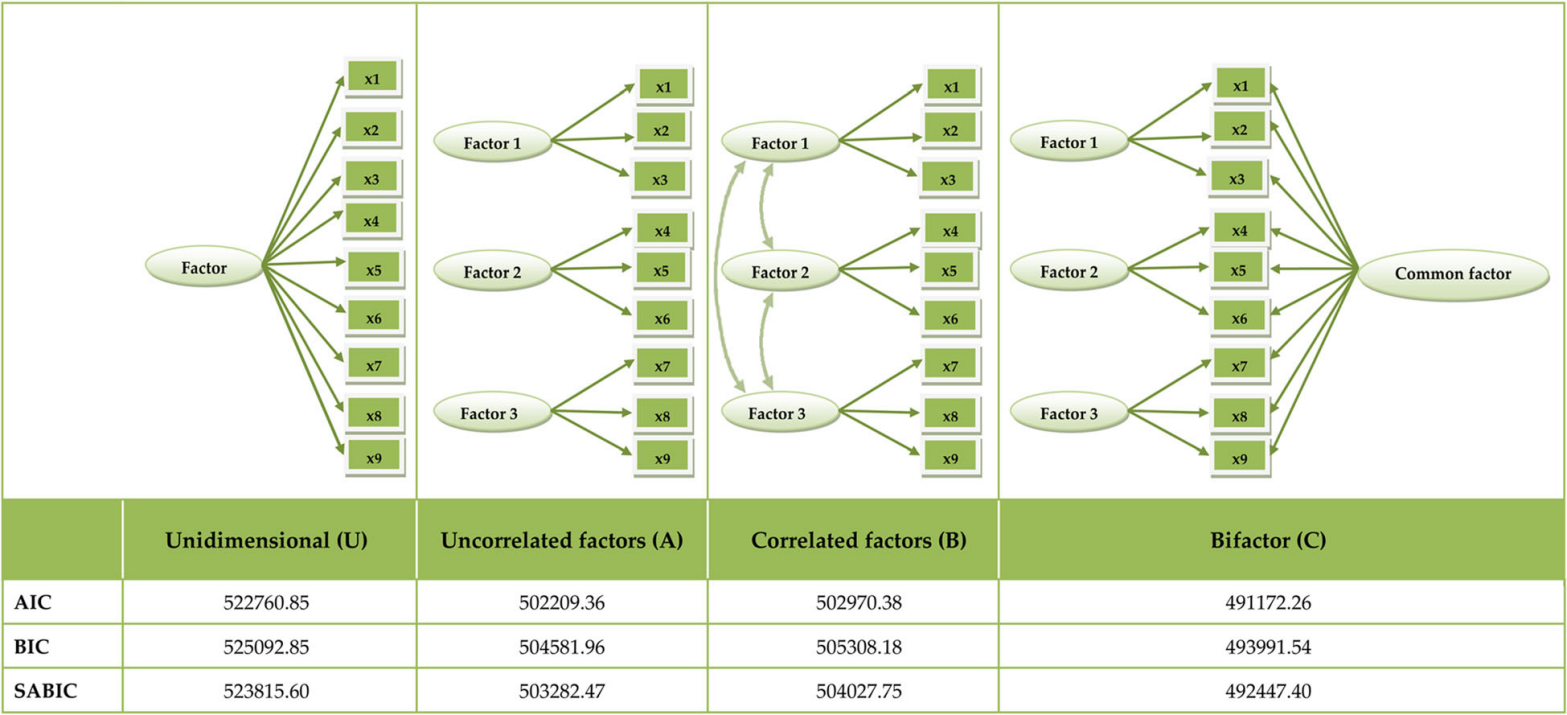
Comparisons of models (schematic picture and the results of obtained fit indices in the table beneath)

The unidimensional CFA showed significant factor loadings for all but 8 items. AIC of the unidimensional model (U) was higher than that of the bifactor (model C), thus supporting the extraction of specific factors in addition to the general factor. We subsequently computed a series of EFAs with WLSMV estimator extracting 1 to 10 factors [40, 41], treating items as categorical variables. The model with 4 factors yielded RMSEA = .042, showing the highest replicability of this factorial solution [35]. As shown in Fig. 1, fit indexes suggested that model C (bifactor with one general and four specific factors) was better than both model A (orthogonal factors) and model B (correlated factors), thus supporting Hypothesis 1. Omega hierarchical was ω_H=_0.80 (see Additional file 1 for the description of the computational procedure). This indicated high saturation of the common variance with the general trait. The results on validity and reliability of measurement (as well as reported in the Additional file 1 age and gender effects) are grouped below under each factor heading.

### General factor (GF)

**Fig. 2:**
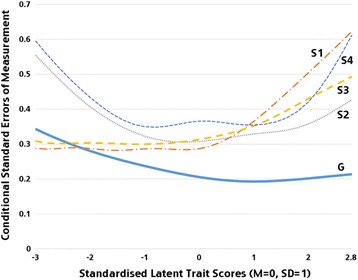
IRT Results for bifactor CFA with one general and 4 specific factors labelled as s1-s4

The general factor (GF) contained high-loading items measuring low agreeableness and high antagonism (*People sometimes find me aloof and distant*), and related to these, lack of social trust (*I feel I have to be on my guard even with friends*), lack of care for others (*I seem very cold and uncaring to others*), and being manipulative of others (*You use or con other people to get what you want*). Moreover, this factor included markers of social avoidance (*I feel very uneasy talking to people I do not know well*) and low communication skills (*I find it hard to communicate clearly what I want to say to people*), (see Additional file 1). Of the 116 items, 108 had statistically significant loadings on this factor (the remaining 8 had low loadings on all factors) and most of the loadings were above .25. Items with high (.60 and above) loadings on this factor came mainly from the Schizotypal Personality Questionnaire, suggesting a significant contribution of schizotypal cognitions (hostile social perceptions and appraisals, low social trust, social avoidance) to the general factor. The coefficient omega for general factor was .95 suggesting very high internal consistency reliability, in agreement with the measurement error curve (Fig. 2) showing low measurement error across a broad range of scores (ranging from + 3 SD to − 3 SD) on this latent dimension. We interpreted this factor as a severity factor, with higher scores indexing more impairment in social functioning.

### Specific factor 1 (SF1)

This factor comprised 20 items, which gauged pro-social affect (e.g., *Do you care about other people’s feelings*) and negative affect such as *“Are you easily embarrassed?”* or *“Do you get upset easily”*, with positive loadings (see Additional file 1). Although the coefficient omega specific ω_S_ = .41 suggested moderate internal consistency, the content of these items (pro-social affect combined with negative affect) suggested a limited theoretical interpretability of this factor. The conditional standard errors curve (Fig. 2) showed high measurement error in the upper range of scores (above + 1 SD) on this latent dimension.

### Specific factor 2 (SF2)

This factor comprised 18 items measuring risk taking and sensation seeking, with positive loadings (e.g., *Do you enjoy things that are risky or dangerous?*) along with items measuring social anxiety, with negative loadings (e.g., *I feel uneasy talking to people I do know well*). The coefficient ω_S_ = 0.35 suggested low internal consistency, in agreement with the conditional standard errors curve, that suggested higher measurement errors when scores were outside of the average range (above + 1 SD or below -SD), with slightly better precision of measurement on the higher than lower end of this factor (Fig. 2).

### Specific factor 3 (SF3)

This factor comprised 24 items with high positive loadings related to effortful control (e.g., *Do you try to do excellent work in school or at work?),* self-direction (*I am self-controlled*) and impulsivity, with negative loadings (*I don’t pay attention*). The omega specific coefficient ω_S_ = .33 suggested low internal consistency; the conditional standard errors curve (Fig. 2) showed better precision of measurement in the low and normal range of scores, but higher measurement error in the higher range of scores (above + 1 SD) on this latent dimension.

### Specific factor 4 (SF4)

This factor was formed of 12 items measuring various aspects of suspicion of others (e.g., *I do not show my emotions to others*; *I feel I have to be on my guard even with friends*), and disorganized thoughts and behaviour (*I find it hard to communicate clearly what I want to say to people, I am an odd, unusual person*)*.* The omega specific coefficient ω_S_ = 0.36 suggested low internal consistency; the conditional standard errors curve showed slightly higher measurement error of this factor compared to other factors. The measurement error was particularly high when scores on this factor were within the very high and very low range (above + 2 SD or below – 2 SD) (Fig. 2).

### Validity

The findings show, overall, statistically significant associations between all factors and validity measures in the expected directions. All correlations were significant and relatively high for GF, and relatively low for specific factors SF 1, 2, 4, and mostly negligible for SF3 (see Table 1). Lower self-esteem and well-being measures, higher depression and anxiety and less positive experience of parenting, as well as the history of self-harm and emotional problems are associated with higher GF scores (higher severity of impairment in social functioning). These results suggest good psychometric validity of the general factor and low validity of the specific factors.

**Table 1:**
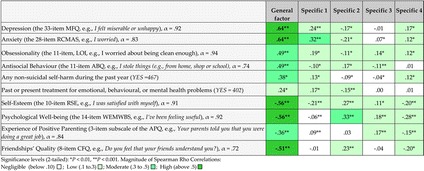
Validation of obtained factors

## Discussion

This study investigated the factor structure of measures of personality traits that were selected because of their known value in revealing characteristics associated with personality difficulties that engage socially disruptive and idiosyncratic behaviours, disorganised thinking and extreme (high or low) emotional style. We hypothesised that distinct personality traits (negative emotionality, antisocial, schizotypal, impulsivity, narcissism, callousness) are underlined by a general latent factor and specific factors. The findings showed support for this hypothesis. We propose that the general latent trait underpinning these traits is best interpreted as a severity factor in which high scores index higher impartment in social functioning whereas low scores index better social functioning. We also suggest that an individual’s locality on this latent trait may index a general liability for risk toward, or resilience away from mental illnesses and the emergence of personality disorders in adulthood. This finding is in line with ICD-11, which suggest an explicit link between personality disorders and compromised interpersonal or social function [42] and supports DSM-5 Section III approach proposing an overarching *dimension of personality functioning* describing one’s relation to the self and others [20]. The present results are also consistent with a study by Sharp and colleagues [18], who identified a general factor that underpinned categorical diagnoses of PD and that of Caspi and colleagues [19] who showed that vulnerability to mental disorder was more convincingly described by a bi-factor model comprising a general psychopathology factor (labelled “p”) and three spectral factors (internalizing, externalizing, and thought disorder), rather than by the spectral factors alone. Individuals who scored highly on the general psychopathology dimension in their study were characterized by “difficulties in regulation/control when dealing with others, the environment, and the self” [19].

As well as the general latent factor common to all the measures, the bifactor model created specific factors from residual variance which are termed specific, as they are independent from the general factor and each other. The general factor demonstrated high reliability and validity as well as low measurement error. The correlations with other measures did not reveal much reliable signal for the specific factors. The evidence from the content and validity analysis of specific factors suggests that they have limited interpretability as meaningful psychological latent constructs and have limited measurement precision (low internal consistency and high measurement error), thus should be interpreted as residual factors. In agreement with psychometric properties of bifactor models reported elsewhere [39], little reliable variance remained in our model beyond that which has been accounted for by the general factor.

### Limitations

Main limitations of the present study include volunteer sampling, which may have entailed self-selection bias, and the lack of control for social desirability effects. We did not use measures specifically designed to assess dimensions proposed by DSM-5 Section III. However, we aimed to assess traits corresponding to concepts well-established in the literature and in clinical practice. Also, computational bias favouring fit indices in bifactor models over correlated-factors and higher-order models has been reported in one study based on Monte Carlo Simulations [43]. Finally, we do not know whether these findings from a community sample would be replicated in a clinical sample.

## Conclusions

The present results showing that putatively distinct personality traits are underpinned by one latent trait, imply that the conceptualisation of personality disorders in terms of a graded continuum of liability to mental health illness is more accurate than the conceptualisation in terms of discrete categories or distinct traits. In sum, the present findings provided support for the proposed in DSM-5 Section III the overarching *dimension of personality functioning* and for Research Domain Criteria, which suggest the approach integrating cognition with social processes, arousal/regulatory systems and affective functions as the major dimensions underlying personality disorders [44, 45]. Future research may reveal longitudinal associations between scores on the latent severity factor and risk for mental illness and personality disorders; this would test the replicability of the findings in clinical populations and explore the usefulness of the latent severity factor in clinical practice.

## Additional file

Additional file 1: Online Supplement. (PDF 3750 kb)

## Abbreviations

AIC: Akaike Information Criterion
BIC: Bayesian Information Criterion
CFA: Confirmatory Factor Analysis
EFA: Exploratory Factor Analysis
IRT: Item Response Theory
RMSEA: Root Mean Square Error of Approximation
SABIC: Sample-Adjusted Bayesian Information Criterion
WLSMV: Weighted Least Squares Mean and Variance-adjusted estimator
