# Supplementary material for: Personality dimensions emerging during adolescence and young adulthood are underpinned by a single latent trait indexing impairment in social functioning
Source: BMC Psychiatry. 2018 Jan 26;18:23. doi: 10.1186/s12888-018-1595-0 (PMC5787243; doi:10.1186/s12888-018-1595-0)
Supplement: Additional file 1: — Online Supplement. (PDF 3750 kb) [file 12888_2018_1595_MOESM1_ESM.pdf]

Online Supplement

The Antisocial Process Screening Device (APSD)

Callous-Unemotional Scale:

APSD03: You care about how well you do at school/work. (R)

APSD07: You are good at keeping promises. (R)

APSD12: You feel bad or guilty when you do something wrong. (R)

APSD18: You are concerned about the feelings of others. (R)

APSD19: You hide your feelings or emotions from others.

Impulsivity:

APSD01: You blame others for your mistakes.

APSD04: You act without thinking of the consequences.

APSD09: You get bored easily.

APSD13: You do risky or dangerous things.

APSD17: You do not plan ahead or you leave things until the last minute.

Narcissism:

APSD05: Your emotions are shallow and fake.

APSD08: You brag a lot about your abilities, accomplishments, or possessions.

APSD10: You use or con other people to get what you want.

APSD11: You tease or make fun of other people.

APSD14: You act charming and nice to get things you want.

APSD15: You get angry when corrected or punished.

APSD16: You think you are better or more important than other people.

The Barratt Impulsiveness Scale (BIS)

Attentional Impulsivity:

BIS05: I don't pay attention.

BIS06: I have racing thoughts.

BIS09: I concentrate easily. (R)

BIS11: I squirm at plays and lectures.

BIS20: I am a steady thinker. (R)

BIS26: I often have extraneous thoughts when thinking.

BIS28: I am restless at the theatre or lecture.

Motor Impulsivity:

BIS02: I do things without thinking.

BIS04: I am happy-go-lucky.

BIS17: I act on impulse.

BIS19: I act on the spur of the moment.

BIS22: I buy things on impulse.

BIS25: I spend or charge more than I earn.

BIS30: I am future orientated. (R)

Non-planning Impulsivity:

BIS01: I plan tasks carefully. (R)

BIS07: I plan trips well ahead of time. (R)

BIS08: I am self controlled. (R)

BIS10: I save regularly. (R)

BIS12: I am a careful thinker. (R)

BIS13: I plan for job security. (R)

BIS14: I say things without thinking.

BIS15: I like to think about complex problems. (R)

BIS18: I get easily bored when solving thought problems.

BIS27: I am more interested in the present than the future.

BIS29: I like puzzles. (Reversed)

The Child and Adolescent Dispositions Scale (CADS)

Daring:

CADS03: Are you daring and adventurous?

CADS06: Do you like rough games and sports?

CADS11: Do you like things that are exciting and loud?

CADS50: Are you brave?

Negative Emotionality:

CADS20: Are you emotional?

CADS23: Are you easily embarrassed?

CADS28: Do you get upset easily?

CADS36: Do you react intensely when you get upset?

CADS40: Are you selfish?

CADS44: Are you calm and easy-going? (R)

CADS46: Do you exaggerate things and blow them out of proportion?

CADS47: Are you jealous of what other people have?

CADS49: Do your moods change unpredictably?

Prosociality:

CADS04: Do you do things to help other people your age without being asked?

CADS05: Do you try to do excellent work in school or at work?

CADS07: Would you feel guilty if you did something that broke the law?

CADS17: Do you share your things with other people without being asked?

CADS19: Do you feel bad for other people when they get hurt?

CADS34: Do you try to cheer up other people your age who are sad or upset?

CADS37: Do you feel sorry for kids who get picked on?

CADS38: Are you cautious?

CADS41: Do you want everyone to follow the rules, including yourself?

CADS42: Do you care about other people's feelings?

CADS43: Do you enjoy learning about new and interesting things?

CADS48: Are you concerned about what is right and wrong?

The Inventory of Callous and Unemotional Traits (ICU)

Callousness:

ICU02: What I think is right and wrong is different from what other people think.

ICU04: I do not care who I hurt to get what I want.

ICU07: I do not care about being on time.

ICU08: I am concerned about the feelings of others. (R)

ICU09: I do not care if I get into trouble.

ICU10: I do not let my feelings control me.

ICU11: I do not care about doing things well.

ICU12: I seem very cold and uncaring to others.

ICU20: I do not like to put the time into doing things well.

ICU21: The feelings of others are unimportant to me.

Uncaring:

ICU03: I care about how well I do at school or work.

ICU05: I feel bad or guilty when I do something wrong.

ICU13: I easily admit to being wrong.

ICU15: I always try my best.

ICU16: I apologise (say I am sorry) to persons I hurt.

ICU17: I try not to hurt others feelings.

ICU23: I work hard on everything I do.

ICU24: I do things to make others feel good.

Unemotional:

ICU01: I express my feelings openly.

ICU06: I do not show my emotions to others.

ICU14: It is easy for others to tell how I am feeling.

ICU19: I am very expressive and emotional.

ICU22: I hide my feelings from others.

Cognitive-Perceptual:

SPQ13: Have you ever had the sense that some person or force is around you, even though you cannot see anyone?

SPQ21: Are you sometimes sure that other people can tell what you are thinking?

SPQ28: Have you ever noticed a common event or object that seemed to be a special sign for you?

SPQ44: Do you often pick up hidden threats or put-downs from what people say or do?

SPQ45: When shopping do you get the feeling that other people are taking notice of you?

SPQ47: Have you had experiences with astrology, seeing the future, UFOs, ESP, or a sixth sense?

SPQ61: Do you ever suddenly feel distracted by distant sounds that you are not normally aware of?

SPQ65: Do you often have to keep an eye out to stop people from taking advantage of you?

Disorganized:

SPQ14: People sometimes comment on my unusual mannerisms and habits.

SPQ32: Some people think that I am a very bizarre person.

SPQ42: Some people find me a bit vague and elusive during a conversation.

SPQ67: I am an odd, unusual person.

SPQ69: I find it hard to communicate clearly what I want to say to people.

Interpersonal:

SPQ08: People sometimes find me aloof and distant.

SPQ36: I feel I have to be on my guard even with friends.

SPQ46: I feel very uncomfortable in social situations involving unfamiliar people.

SPQ52: Have you found that it is best not to let other people know too much about you?

SPQ57: I tend to keep in the background on social occasions.

SPQ66: Do you feel that you cannot get close to people?

SPQ71: I feel very uneasy talking to people I do not know well.

SPQ73: I tend to keep my feelings to myself.

Analytical strategy

The following formulae were used to compute omega coefficients[1,2] ω_H (indicating how much common variance is accounted for by the general trait), ω_G (indicating the internal consistency reliability of the general factor when controlling for the effects of all specific factors), ω_S (indicating the internal consistency reliability of a specific factor controlling for the effect of other factors in the model):

$$\omega_G = \frac{(\sum \lambda_{iGEN})^2}{(\sum \lambda_{iGEN})^2 + \sum \theta_i}$$

$$\omega_S = \frac{(\sum \lambda_{iS})^2}{(\sum \lambda_{iS})^2 + (\sum \lambda_{iGEN/S})^2 + \sum \theta_i}$$

$$\omega_H = \frac{(\sum \lambda_{iGEN})^2}{(\sum \lambda_{iGEN})^2 + (\sum \lambda_{iS_1})^2 ..... + (\sum \lambda_{iS_P})^2 + \sum \theta_i}$$

λ_{iGEN} factor loadings on the General Factor

λ_{iS} factor loadings on a Specific Factor

$\lambda_{iGEN/S}$ factor loadings of the General Factor including only items making up a given Specific Factor S

θ_i items' unique (error) variances

Prior to computing ω_H and ω_G items with negative loadings on the general factor were reversed and factor loadings from the subsequent bifactor CFA were used in these formulae[1]; absolute values of factor loadings were used to compute ω_S [1].

Results

Gender and Age Effects

General Factor (GF)

This factor showed significant main effects of age (F(4, 2277)=6.07, p< .001, η^2=.010, adolescents > adults, the mean difference for age groups 16-19 and 22-24 =. 23, p<.001) and gender (F(1, 2277)=7.70, p<.006, η^2=.003, boys > girls, the mean difference .11, p<.006). There was an interaction between age and gender (F(4, 2277)=3.92, p< .003, η^2=.006) indicating faster decrease in GF in older age groups in girls compared to boys (see Supplementary Figure 2).

Specific factor 1 (SF1)

There were main effects of gender (F(1, 2277)= 262.27, p< .001, η^2=.10, girls > boys, mean difference = .58, p<.001, see Supplementary Figure 2) and of age (F(4, 2277)= 6.46, p<.001, η^2=.011; adults > adolescents, mean difference between 14-15 and 22-24 =.24, p<.001), the correlation (Spearman's rho) between age and this factor was however negligible (.09, p<.001).

Specific factor 2 (SF2)

There was a main effect of gender (F(1, 2277 = 159.23, p <.001, η^2 = .06 ; males > females, mean diffrence.45, p<.001). No significant main effect of age, or gender x age interaction was found (see Supplementary Figure 2).

Specific factor 3 (SF3)

There was a main effect of age ($F(4, 2277) = 8.18$, $p <.001$, $\eta^2 = .014$, older > younger, mean difference between 14-15 and 22-24 years old, 0.20, $p<.005$). The main effect of gender was marginal ($\eta^2 =.003$; girls > boys .09, $p=.014$). There was no age x gender interaction (see Supplementary Figure 2).

Specific factor 4 (SF4)

There was a significant main effect of age ($F(4, 2277)=4.38$, $p=.002$, $\eta^2 =.009$); 18-19>14-15 (mean difference .18, $p = .023$) and 18-19>22-14 (mean difference .18, $p=.012$) and significant main effect of gender ($F(4, 2277)= 12.73$, $p<.001$, $\eta^2 =.006$); boys >girls .13, $p<.001$. There was an age x gender interaction ($F(4, 2277) = 3.36$, $p =.009$, $\eta^2 =.006$); overall females had lower scores than males in all age groups (in particular 22-24), except for the group 14-15, where this trend was reversed for the genderes (see Supplementary Figure 2).

References (Supplement):

1. Gignac, G.E. On the inappropiatness of using items to calculate total scle score reliability via coefficient Alpha for multidimensional scales. *Eur J Psychol Assess.* **30**, 130-139 (2014).

2. Reise, S. P. The rediscovery of bifactor measurement models. *Multivariate Behav Res.* **47**, 667-696 (2012).

Figure 1. Network plot of correlations between 116 items used in this study (represented as circles)

Positive correlations are shown as green lines; negative as red lines. Only correlations of .25, or above are depicted in the graph. The thicker the line, the higher is the correlation. The number of an item corresponds to that one in the Table with factor loadings reported on pages 4–9 of this Supplement.

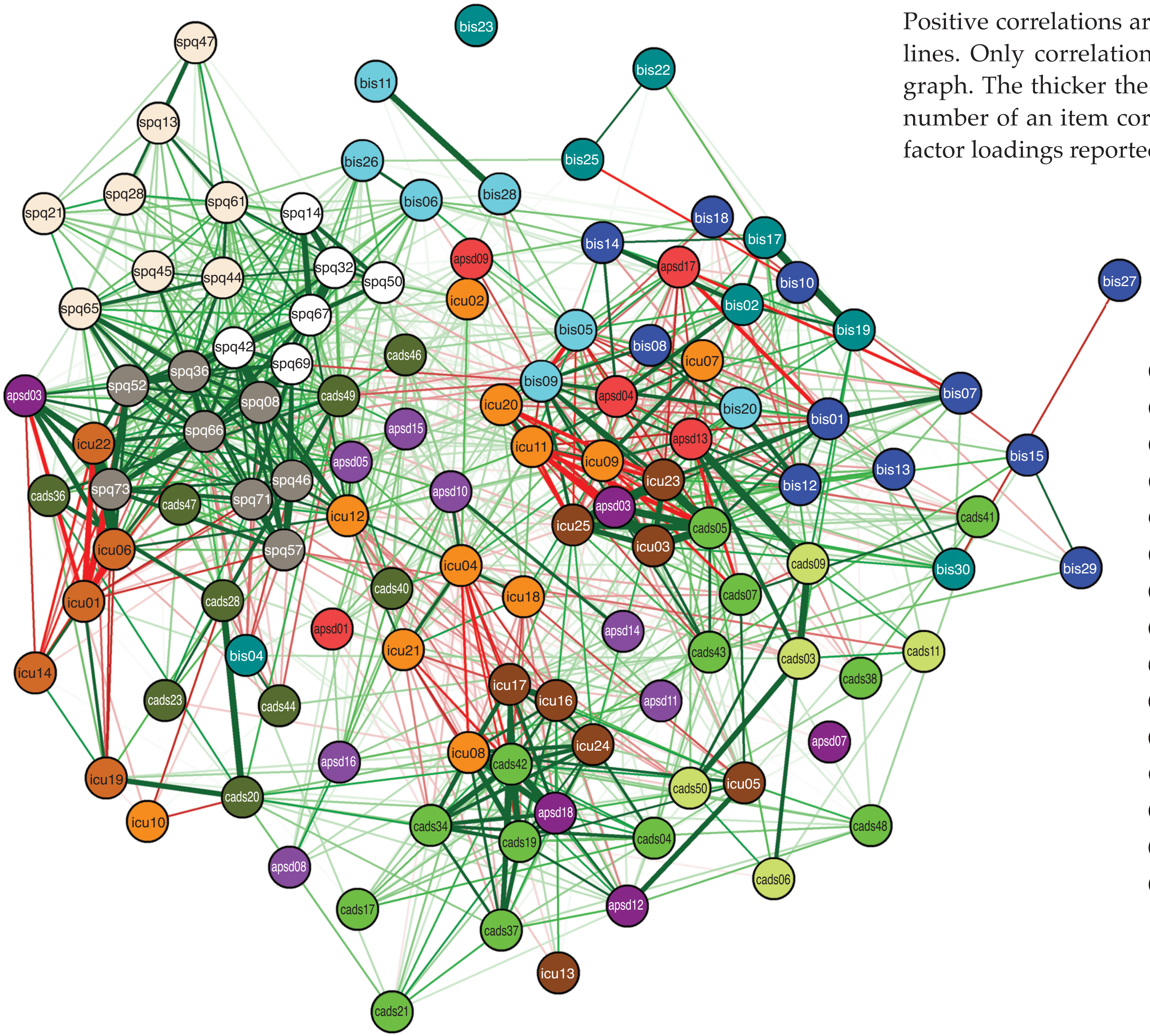

Figure 2. Age and Gender Effects on Levels of the General Factor and Specific Factors (with confidence intervals)

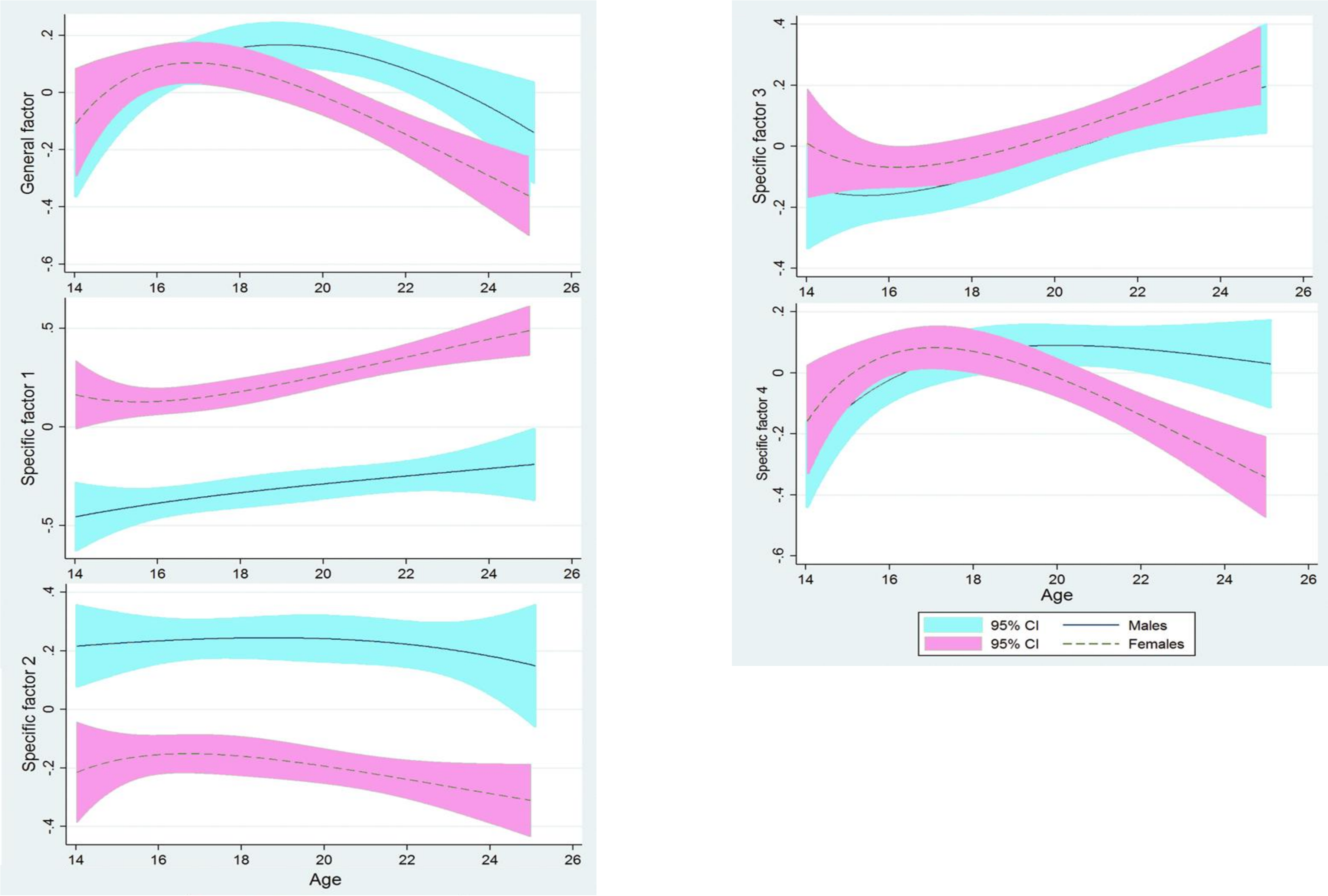

Table 1. Factor Loadings in bifactor EFA and CFA

ITEM NR	SCALE	ITEM	EFA-GF	EFA-SF1	EFA-SF2	EFA-SF3	EFA-SF4	CFA-GF	CFA-SF1	CFA-SF2	CFA-SF3	CFA-SF4
APSD01	Impulsivity	*You blame others for your mistakes.*	.42*	-.13*	-.00	.07*	-.20*	.40				
APSD03	Callous-Unemotional	*You care about how well you do at school/work.*	-.39*	.14*	-.03	**.67***	-.13*	-.44			**.68**	
APSD04	Impulsivity	*You act without thinking of the consequences.*	.50*	-.08*	.28*	-.22*	-.11*	**.58**				
APSD05	Narcissism	*Your emotions are shallow and fake.*	**.52***	-.12*	.04	.03	.19*	**.55**				
APSD07	Callous-Unemotional	*You are good at keeping promises.*	-.26*	.20*	.14*	.13*	.15*	-.30				
APSD08	Narcissism	*You brag a lot about your abilities, accomplishments, or possessions.*	.22*	-.18*	.18*	.24*	-.24*	.17				
APSD09	Impulsivity	*You get bored easily.*	.48*	.04	.06*	-.09*	.01	.49				
APSD10	Narcissism	*You use or con other people to get what you want.*	**.54***	-.17*	.22*	.16*	-.12*	**.51**				
APSD11	Narcissism	*You tease or make fun of other people.*	.31*	-.22*	.14*	.14*	-.17*	.30				
APSD12	Callous-Unemotional	*You feel bad or guilty when you do something wrong.*	-.01	**.57***	-.08*	.18*	.00	-.08	**.59**			
APSD13	Impulsivity	*You do risky or dangerous things.*	.39*	-.05*	**.66***	-.05*	.06*	.43		**.65**		
APSD14	Narcissism	*You act charming and nice to get things you want.*	.28*	-.03	.27*	.18*	-.21*	.23				
APSD15	Narcissism	*You get angry when corrected or punished.*	**.53***	.04	-.03	.06*	-.22*	.48				
APSD16	Narcissism	*You think you are better or more important than other people.*	.31*	-.22*	.11*	.35*	-.22*	.30			.26	
APSD17	Impulsivity	*You do not plan ahead or you leave things until the last minute.*	.36*	.03	.19*	-.40*	.03	.38			-.40	
APSD18	Callous-Unemotional	*You are concerned about the feelings of others.*	-.26*	**.71***	.06*	.03	.01	-.27	**.71**			
APSD19	Callous-Unemotional	*You hide your feelings or emotions from others.*	.40*	.12*	.00	.00	**.57***	.36				**.63**
BIS01	Nonplanning-Impulsivity	*I plan tasks carefully.*	-.30*	.10*	-.13*	**.57***	.02	-.33			**.57**	
BIS02	Motor-Impulsivity	*I do things without thinking.*	.43*	.02	.35*	-.25*	-.17*	.49		.34		

Significance levels (2-tailed): *$P < 0.01$. Magnitude of Loadings: Negligible (below .10) □, Low (.1 to.3) □, Moderate (above .3 to .5) □, High (above .5) ■.

ITEM NR	SCALE	ITEM	EFA-GF	EFA-SF1	EFA-SF2	EFA-SF3	EFA-SF4	CFA-GF	CFA-SF1	CFA-SF2	CFA-SF3	CFA-SF4
BIS04	Motor-Impulsivity	*I am happy-go-lucky.*	-.28*	.05*	.46*	-.02	-.16*	-.29		.45		
BIS05	Attentional-Impulsivity	*I don't pay attention.*	**.52***	-.02	.13*	-.35*	-.04	**.54**			-.30	
BIS06	Attentional-Impulsivity	*I have racing thoughts.*	**.53***	.17*	.17*	.04	.01	.50				
BIS07	Nonplanning-Impulsivity	*I plan trips well ahead of time.*	-.18*	.10*	-.16*	.49*	-.02	-.21			.50	
BIS08	Nonplanning-Impulsivity	*I am self-controlled.*	-.39*	-.01	.08*	.40*	.14*	-.38			.39	
BIS09	Attentional-Impulsivity	*I concentrate easily.*	**-.54***	-.05*	.06*	.48*	.00	**-.54**			.45	
BIS10	Nonplanning-Impulsivity	*I save regularly.*	-.34*	-.02	-.05*	.36*	.09*	-.34			.34	
BIS11	Attentional-Impulsivity	*I squirm at plays and lectures.*	.37*	.04	.03	-.03	.04	.38				
BIS12	Nonplanning-Impulsivity	*I am a careful thinker.*	-.34*	.12*	-.03	**.54***	.13*	-.36			**.52**	
BIS13	Nonplanning-Impulsivity	*I plan for job security.*	-.22*	.09*	-.01	.47*	-.02	-.25			.47	
BIS14	Nonplanning-Impulsivity	*I say things without thinking.*	.47*	.03	.24*	-.11*	-.20*	.49				
BIS15	Nonplanning-Impulsivity	*I like to think about complex problems.*	.01	.06*	.25*	.47*	.08*	.00			.45	
BIS17	Motor-Impulsivity	*I act on impulse.*	.38*	.10*	**.53***	-.09*	-.19*	.39		**.55**		
BIS18	Nonplanning-Impulsivity	*I get easily bored when solving thought problems.*	.33*	-.00	-.03	-.29*	-.09*	.40				
BIS19	Motor-Impulsivity	*I act on the spur of the moment.*	.31*	.10*	**.59***	-.14*	-.19*	.33		**.62**		
BIS20	Attentional-Impulsivity	*I am a steady thinker.*	-.37*	-.01	.07*	.50*	.11*	-.37			.49	
BIS22	Motor-Impulsivity	*I buy things on impulse.*	.27*	.18*	.15*	-.06*	-.19*	.25				

Significance levels (2-tailed): **P* < 0.01. Magnitude of Loadings: Negligible (below .10) ☐, Low (.1 to.3) ☐, Moderate (above .3 to .5) ☐, High (above .5) ■.

ITEM NR	SCALE	ITEM	EFA-GF	EFA-SF1	EFA-SF2	EFA-SF3	EFA-SF4	CFA-GF	CFA-SF1	CFA-SF2	CFA-SF3	CFA-SF4
BIS23	Motor-Impulsivity	*I can only think about one thing at a time.*	.23*	.01	-.08*	-.08*	.03	.25				
BIS25	Motor-Impulsivity	*I spend or charge more than I earn.*	.40*	.09*	.11*	-.13*	-.14*	.40				
BIS26	Attentional-Impulsivity	*I often have extraneous thoughts when thinking.*	.47*	.16*	.12*	.10*	.06	.43				
BIS27	Nonplanning-Impulsivity	*I am more interested in the present than the future.*	.07*	-.02	.21*	-.23*	.00	.15				
BIS28	Attentional-Impulsivity	*I am restless at the theatre or lecture.*	.45*	.02	.07*	-.10*	.03	.48				
BIS29	Nonplanning-Impulsivity	*I like puzzles.*	-.13*	.06*	.12*	.34*	.11*	-.13			.34	
BIS30	Motor-Impulsivity	*I am future orientated.*	-.11*	.11*	.02	**.54***	-.08*	-.16			**.51**	
CADS03	Daring	*Are you daring and adventurous?*	.07*	.03	**.77***	.08*	-.02	.06		**.78**		
CADS04	Prosociality	*Do you do things to help other people your age without being asked?*	-.19*	.47*	.32*	.19*	.04*	-.24	.45			
CADS05	Prosociality	*Do you try to do excellent work in school or at work?*	-.36*	.21*	-.02	**.70***	-.07*	-.41			**.71**	
CADS06	Daring	*Do you like rough games and sports?*	.00	-.19*	**.52***	.12*	.07*	.01		**.53**		
CADS07	Prosociality	*Would you feel guilty if you did something that broke the law?*	-.32*	.31*	-.33*	.21*	.04*	-.44		-.33		
CADS09	Daring	*Do you enjoy doing things that are risky or dangerous?*	.25*	-.10*	**.77***	.00	.06*	.28		**.78**		
CADS11	Daring	*Do you like things that are exciting and loud?*	.04	.07*	.49*	-.06*	-.17*	.04		**.51**		
CADS17	Prosociality	*Do you share your things with other people without being asked?*	-.18*	.38*	.23*	.05*	-.11*	-.20	.37			
CADS19	Prosociality	*Do you feel bad for other people when they get hurt?*	-.21*	**.72***	-.06*	.05*	-.02	-.25	**.73**			
CADS20	Negative Emotionality	*Are you emotional?*	.16*	**.61***	-.26*	.00	-.39*	.11	**.63**			
CADS21	Prosociality	*Would you get upset if you saw an animal being hurt?*	-.03	.46*	-.12*	-.01	-.09*	-.04	.48			

Significance levels (2-tailed): *$P < 0.01$. Magnitude of Loadings: Negligible (below .10) □, Low (.1 to.3) □, Moderate (above .3 to .5) □, High (above .5) □.

ITEM NR	SCALE	ITEM	EFA-GF	EFA-SF1	EFA-SF2	EFA-SF3	EFA-SF4	CFA-GF	CFA-SF1	CFA-SF2	CFA-SF3	CFA-SF4
CADS23	Negative Emotionality	*Are you easily embarrassed?*	.25*	.29*	-.39*	.03	.07	.20		-.40		
CADS28	Negative Emotionality	*Do you get upset easily?*	.43*	**.51***	-.39*	.01	-.21*	.41	**.58**			
CADS34	Prosociality	*Do you try to cheer up other people your age who are sad or upset?*	-.22*	**.66***	.21*	.07*	-.09*	-.25	**.64**			
CADS36	Negative Emotionality	*Do you react intensely when you get upset?*	.46*	.29*	-.15*	.10*	-.26*	.37				
CADS37	Prosociality	*Do you feel sorry for kids who get picked on?*	-.09*	**.63***	-.04*	.05*	-.01	-.11	**.64**			
CADS38	Prosociality	*Are you cautious?*	-.03	.26*	-.37*	.30*	.09*	-.15		-.39		
CADS40	Negative Emotionality	*Are you selfish?*	.47*	-.19*	-.08*	.09*	-.18*	.45				
CADS41	Prosociality	*Do you want everyone to follow the rules, including yourself?*	-.18*	.14*	-.28*	.34*	.08*	-.24			.29	
CADS42	Prosociality	*Do you care about other people's feelings?*	-.31*	**.81***	-.00	.01	-.02	-.34	**.81**			
CADS43	Prosociality	*Do you enjoy learning about new and interesting things?*	-.24*	.29*	.22*	.44*	-.02	-.29			.45	
CADS44	Negative Emotionality	*Are you calm and easy-going?*	-.35*	.06*	.42*	.01	.11*	-.34		.35		
CADS46	Negative Emotionality	*Do you exaggerate things and blow them out of proportion?*	.44*	.03	.01	.10*	-.30*	.40				-.19
CADS47	Negative Emotionality	*Are you jealous of what other people have?*	.46*	.11*	-.17*	.04	-.14*	.40				
CADS48	Prosociality	*Are you concerned about what is right and wrong?*	-.06*	.37*	-.11*	.27*	.00	-.16	.39			
CADS49	Negative Emotionality	*Do your moods change unpredictably?*	**.61***	.28*	-.15*	-.05*	-.09	**.57**				
CADS50	Daring	*Are you brave?*	-.05	-.01	**.62***	.17*	-.02	-.10		**.63**		
ICU01	Unemotional	*I express my feelings openly.*	-.31*	.13*	.17*	.06*	**-.67***	-.30				**-.73**
ICU02	Callousness	*What I think is right and wrong is different from what other people think.*	.40*	-.06*	.18*	.13*	-.02	.38				
ICU03	Uncaring	*I care about how well I do at school or work.*	-.37*	.18*	.00	**.68***	-.10*	-.41			**.70**	

Significance levels (2-tailed): *$P < 0.01$. Magnitude of Loadings: Negligible (below .10) □, Low (.1 to.3) □, Moderate (above .3 to .5) □, High (above .5) ■.

ITEM NR	SCALE	ITEM	EFA-GF	EFA-SF1	EFA-SF2	EFA-SF3	EFA-SF4	CFA-GF	CFA-SF1	CFA-SF2	CFA-SF3	CFA-SF4
ICU04	Callousness	*I do not care who I hurt to get what I want.*	**.55***	-.41*	.21*	.20*	-.09*	**.52**	-.38			
ICU05	Uncaring	*I feel bad or guilty when I do something wrong.*	-.17*	**.54***	-.18*	.20*	.04	-.25	**.53**			
ICU06	Unemotional	*I do not show my emotions to others.*	.39*	-.11*	-.00	.02	**.70***	.37				**.74**
ICU07	Callousness	*I do not care about being on time.*	.40*	-.08*	.24*	-.30*	.00	.44			-.27	
ICU08	Callousness	*I am concerned about the feelings of others.*	-.31*	**.75***	-.01	.04	.00	-.33	**.74**			
ICU09	Callousness	*I do not care if I get into trouble.*	.42*	-.16*	.43*	-.24*	.08*	**.54**		.38		
ICU10	Callousness	*I do not let my feelings control me.*	-.19*	-.25*	.26*	.13*	.27*	-.15				
ICU11	Callousness	*I do not care about doing things well.*	**.50***	-.10*	.08*	-.42*	.11*	**.54**			-.41	
ICU12	Callousness	*I seem very cold and uncaring to others.*	**.58***	-.24*	.00	.14*	.27*	**.61**				
ICU13	Uncaring	*I easily admit to being wrong.*	-.24*	.27*	.10*	.01	.15*	-.26				
ICU14	Unemotional	*It is easy for others to tell how I am feeling.*	-.23*	.21*	.05*	.02	-.47*	-.24				**-.53**
ICU15	Uncaring	*I always try my best.*	-.48*	.20*	.06*	**.62***	.03	-.50			**.64**	
ICU16	Uncaring	*I apologise (say I am sorry) to persons I hurt.*	-.38*	**.55***	.05*	.05*	.10*	-.40	**.52**			
ICU17	Uncaring	*I try not to hurt others feelings.*	-.41*	**.62***	.01	.02	.16*	-.42	**.60**			
ICU18	Callousness	*I do not feel remorseful when I do something wrong.*	.39*	-.32*	.19*	-.04	.05	.43	-.32			
ICU19	Unemotional	*I am very expressive and emotional.*	-.01	.43*	-.03	.06*	**-.56***	-.08				**-.54**
ICU20	Callousness	*I do not like to put the time into doing things well.*	.47*	-.08*	.04	-.32*	.03	.48			-.33	
ICU21	Callousness	*The feelings of others are unimportant to me.*	.40*	-.40*	.09*	.07*	.11*	.41	-.38			
ICU22	Unemotional	*I hide my feelings from others.*	.46*	.01	-.02	.04	**.70***	.42				**.74**
ICU23	Uncaring	*I work hard on everything I do.*	-.46*	.16*	.03	**.67***	.03*	-.47			**.69**	
ICU24	Uncaring	*I do things to make others feel good.*	-.30*	**.56***	.17*	.18*	.01	-.36	**.54**			
SPQ08	Interpersonal	*People sometimes find me aloof and distant.*	**.61***	.08*	-.14*	.04	.31*	**.59**				.27
SPQ13	Cognitive-Perceptual	*Have you ever had the sense that some person or force is around you, even though you cannot see anyone?*	.37*	.22*	.06*	.03	.10*	.34				
SPQ14	Disorganized	*People sometimes comment on my unusual mannerisms and habits.*	**.55***	.13*	.10*	.11*	.11*	**.52**				
SPQ21	Cognitive-Perceptual	*Are you sometimes sure that other people can tell what you are thinking?*	.38*	.20*	.00	.08*	.00	.34				

Significance levels (2-tailed): *$P < 0.01$. Magnitude of Loadings: Negligible (below .10) □, Low (.1 to.3) □, Moderate (above .3 to .5) □, High (above .5) □.

ITEM NR	SCALE	ITEM	EFA-GF	EFA-SF1	EFA-SF2	EFA-SF3	EFA-SF4	CFA-GF	CFA-SF1	CFA-SF2	CFA-SF3	CFA-SF4
SPQ28	Cognitive-Perceptual	*Have you ever noticed a common event or object that seemed to be a special sign for you?*	.41*	.18*	.08*	.15*	.00	.35				
SPQ32	Disorganized	*Some people think that I am a very bizarre person.*	**.66***	.15*	.06*	.09*	.12	**.64**				
SPQ36	Interpersonal	*I feel I have to be on my guard even with friends.*	**.66***	.14*	-.17*	.12*	.28*	**.65**				
SPQ42	Disorganized	*Some people find me a bit vague and elusive during a conversation.*	**.58***	.05	-.11*	.05	.31*	**.56**				.27
SPQ44	Cognitive-Perceptual	*Do you often pick up hidden threats or put-downs from what people say?*	**.58***	.17*	.00	.11*	.10	**.54**				
SPQ45	Cognitive-Perceptual	*When shopping, do you get the feeling that other people are taking notice of you?*	.50*	.20*	-.06*	.03	.07	.47				
SPQ46	Interpersonal	*I feel very uncomfortable in social situations involving unfamiliar people.*	**.52***	.11*	**-.54***	-.01	.32*	**.55**		**-.64**		
SPQ47	Cognitive-Perceptual	*Have you had experiences with astrology, seeing the future, UFOs, ESP, or a sixth sense?*	.31*	.13*	.09*	.08*	.08	.28				
SPQ50	Disorganized	*I sometimes use words in unusual ways.*	**.54***	.02	.18*	.17*	.07	.50				
SPQ52	Interpersonal	*Have you found that it is best not to let other people know too much about you?*	**.56***	.05*	-.09*	.09*	.43*	**.52**				.46
SPQ57	Interpersonal	*I tend to keep in the background on social occasions.*	.42*	.04	-.45*	-.03	.40*	.48		**-.53**		
SPQ61	Cognitive-Perceptual	*Do you ever suddenly feel distracted by distant sounds that you are not normally aware of?*	.50*	.13*	.04	.07*	.17*	.49				
SPQ65	Cognitive-Perceptual	*Do you often have to keep an eye out to stop people from taking advantage of you?*	**.51***	.24*	-.05	.14*	.18*	.47				
SPQ66	Interpersonal	*Do you feel that you cannot get close to people?*	**.60***	.06*	-.20*	.06*	.39*	**.57**				.41
SPQ67	Disorganized	*I am an odd, unusual person.*	**.70***	.13*	.03	.12*	.21*	**.68**				
SPQ69	Disorganized	*I find it hard to communicate clearly what I want to say to people.*	**.56***	.09*	-.20*	-.04	.26*	**.59**				
SPQ71	Interpersonal	*I feel very uneasy talking to people I do not know well.*	**.51***	.08*	**-.51***	-.05*	.36*	**.56**		**-.60**		
SPQ73	Interpersonal	*I tend to keep my feelings to myself.*	.45*	-.04	-.15*	.06*	**.72***	.43				**.75**

Significance levels (2-tailed): *$P < 0.01$. Magnitude of Loadings: Negligible (below .10) ☐, Low (.1 to.3) ☐, Moderate (above .3 to .5) ☐, High (above .5) ☐.
